# Transcriptomic Profiling Unveils *EDN3* ^+^ Meningeal Fibroblasts as Key Players in Sturge‐Weber Syndrome Pathogenesis

**DOI:** 10.1002/advs.202408888

**Published:** 2025-02-08

**Authors:** Daosheng Ai, Tianyue Ming, Xiaoli Li, Shu Wang, Zhanying Bi, Jinyi Zuo, Zizhang Cheng, Weijin Sun, Mingguo Xie, Fengzhi Li, Xiongfei Wang, Xueling Qi, Guoming Luan, Woo‐ping Ge, Yuguang Guan

**Affiliations:** ^1^ Academy for Advanced Interdisciplinary Studies (AAIS) Peking University Beijing 100871 China; ^2^ Beijing Institute for Brain Research Chinese Academy of Medical Sciences & Peking Union Medical College Beijing 102206 China; ^3^ Chinese Institute for Brain Research (CIBR) Beijing Beijing 102206 China; ^4^ Department of Neurology Affiliated Zhongda Hospital Southeast University Nanjing 210009 China; ^5^ Department of Neurosurgery SanBo Brain Hospital Capital Medical University Beijing 100093 China; ^6^ College of Life Sciences Nankai University Tianjin 300071 China; ^7^ State Key Laboratory of Cognitive Neuroscience and Learning Beijing Normal University Beijing 100875 China; ^8^ Department of Pathology SanBo Brain Hospital Capital Medical University Beijing 100093 China; ^9^ Beijing Key Laboratory of Epilepsy Beijing 100093 China; ^10^ Center of Epilepsy Beijing Institute of Brain Disorders Collaborative Innovation Center for Brain Disorders Capital Medical University Beijing 100093 China; ^11^ China International Neuroscience Institute Department of Neurosurgery Xuanwu Hospital Beijing Institute of Brain Disorders (BIBD) Capital Medical University Beijing 100053 China

**Keywords:** cerebrovasculature, meningeal fibroblasts, single‐cell RNA sequencing, Sturge‐Weber syndrome

## Abstract

Sturge‐Weber syndrome (SWS) is characterized by leptomeningeal vascular malformation, resulting in significant risks of life‐threatening seizures and strokes. The current absence of specific treatments underscores the need to define the molecular and cellular mechanisms that drive the progression of SWS. Here, the transcriptome of 119 446 cells isolated from both malformed tissues and peri‐lesion tissues from the brains of patients with SWS is examined. This comprehensive analysis finds a complex landscape of cell heterogeneity and distinct cell substate associated with the evolution of this disease are revealed. Notably, a unique fibroblast cluster and molecular mechanism are identified that contribute to the development of SWS. These findings not only expand the understanding of SWS but also open up promising avenues for therapeutic interventions.

## Graphical abstract

Sturge‐Weber syndrome (SWS) is characterized by leptomeningeal vascular malformations, leading to seizures and stroke. Analysis of 119 446 brain cells from SWS patients uncovered distinct cell heterogeneity and identified an *EDN3*⁺ meningeal fibroblast cluster, with *WNT5A* emerging as a potential key driver of SWS progression and a promising therapeutic target.

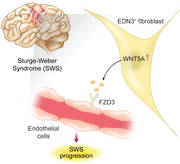

## 1 Introduction

The cerebrovasculature, which is densely and precisely distributed throughout the brain, plays a crucial role in meeting the substantial energy demands required for neurological function. This vascular network is vital for maintaining brain homeostasis and facilitating the transport of biomolecules.^[^ ^1^, ^2^ ^]^ When abnormal vascular growth leads to vascular malformations in the brain or meninges, they can result in acute complications and chronic neurological dysfunction.^[^ ^3^, ^4^ ^]^ Additionally, cerebrovascular malformations occurring in arteries, veins, or capillaries can manifest in diverse clinical presentations, courses, and complication rates.^[^ ^3^ ^]^

Understanding the human cerebrovasculature in various pathological conditions is a pressing need for both scientific research and clinical applications. SWS, a non‐hereditary neurovascular disorder, is characterized by leptomeningeal vascular malformation, facial capillary malformation, and glaucoma.^[^ ^5^ ^]^ This congenital condition, which occurs sporadically, affects roughly 1 in 20 000 to 50 000 live births, irrespective of gender.^[^ ^6^ ^]^ Individuals with SWS constantly face the risk of seizures, stroke, and stroke‐like episodes, as well as motor and cognitive difficulties, leading to significant morbidity and mortality.^[^ ^7^ ^]^ Presently, due to the absence of targeted drug therapies, standard neurologic treatment for SWS focuses on symptom management.^[^ ^5^, ^8^ ^]^

In 2013, a somatic activating variant (c.548G→A, p.Arg183Gln) in the G‐protein subunit alpha q (GNAQ) was initially discovered in 88% of SWS patients using whole‐genome sequencing and amplicon sequencing.^[^ ^6^ ^]^ Subsequently, mutations located in subunit alpha 11 (GNA11)^[^ ^9^, ^10^ ^]^ and subunit beta 2 (GNB2)^[^ ^11^ ^]^ have also been reported with potential associations with SWS. Moreover, a murine model, achieved by conditionally expressing a second GNAQ mutant, GNAQ^Q209L^, in endothelial cells, has demonstrated the ability to induce vascular abnormalities that mirror the characteristic vascular tufts and coagulopathy seen in Kasabach‐Merritt phenomenon.^[^ ^12^ ^]^

In addition to the capillary malformation found in SWS, GNAQ and GNA11 mutations have been identified in other vascular malformations or tumors, including congenital hemangioma, tufted angioma, and Kaposiform hemangioendothelioma.^[^ ^13^, ^14^ ^]^ Notably, these conditions are not closely associated with the central nervous system (CNS). Given the distinctive lesion locations and the frequent occurrence of CNS complications in SWS, a comprehensive explanation cannot be adequately provided solely from the standpoint of somatic mutations in GNAQ and its resulting hyperactivation of downstream pathways.

Studying SWS, particularly the development of high‐risk leptomeningeal vascular malformations and associated lethal brain complications, has been challenging due to its rarity and limited access to patient samples for in‐depth investigations. Additionally, there is currently no available animal model that accurately replicates the CNS symptoms observed in SWS patients. In this study, our aim was to directly examine the cellular and molecular mechanisms underlying the exacerbation of SWS in brain vascular malformations.

To achieve this, we obtained samples from malformed SWS lesions and peri‐lesion tissues for comparison during neurosurgery procedures on patients diagnosed with SWS. Using single‐cell RNA sequencing (scRNA‐seq), we conducted a comprehensive analysis of vascular cells, as well as surrounding neurons, glia, and immune cell populations, to gain insights into the cellular mechanisms of SWS. Further, we identified distinct perivascular fibroblast clusters and explored the functional roles of meningeal fibroblasts in the progression of SWS. To uncover factors contributing to the aggravation of SWS vascular malformation and other lethal CNS complications, we constructed a single‐cell atlas of SWS vascular malformation and found differences in patterns of cellular interactions and in transcriptional changes throughout the development of SWS. Our study provides a valuable contribution to understanding the intricate cellular and molecular dynamics involved in the development of SWS, shedding light on potential targets for future therapeutic interventions.

## 2 Results

### 2.1 A Large Single‐Cell RNA Sequencing Atlas to Study Transcriptomic Changes of CNS during SWS Progression

**Figure 1:**
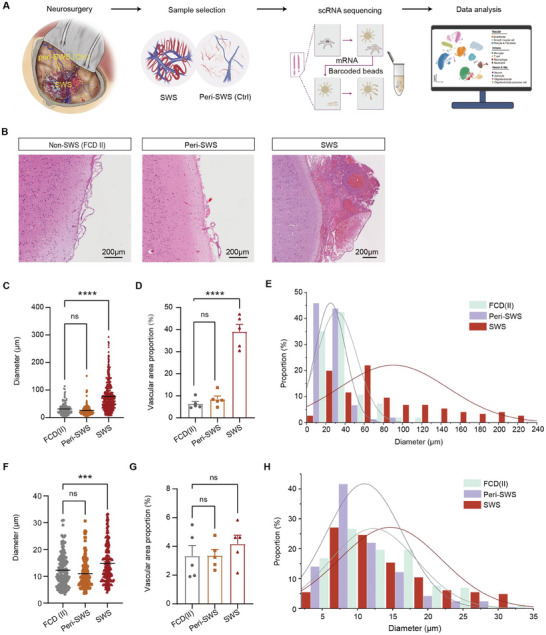
Collection of SWS lesions and peri‐lesion brain tissue from SWS patients. A) Schematic representation of specimen isolation and single‐cell RNA sequencing (scRNA‐seq) processing. B) Hematoxylin and eosin (HE) staining of meningeal and cortical tissue from non‐SWS (FCD II), peri‐SWS, and SWS samples. The SWS sample shows malformed meningeal vessels. Scale bar: 200 µm. C) Comparison of leptomeningeal vascular diameter among SWS lesion areas, peri‐SWS areas, and FCD II lesion areas (non‐SWS). **** *p* < 0.0001, ordinary one‐way ANOVA. Error bars indicate SEM. D) Comparison of leptomeningeal vascular density (area proportion) among SWS lesion areas, peri‐SWS areas, and FCD II lesion areas (non‐SWS). **** *p* < 0.0001, ordinary one‐way ANOVA. All error bars indicate SEM. E) Comparison of leptomeningeal vascular diameter distribution among SWS lesion areas, peri‐SWS areas, and FCD II lesion areas (non‐SWS). F) Comparison of layer 1–2 vascular diameter among SWS lesion areas, peri‐SWS areas, and FCD II lesion areas (non‐SWS). **** *p* < 0.0001, ordinary one‐way ANOVA. Error bars indicate SEM. G) Comparison of layer 1–2 vascular density (area proportion) among SWS lesion areas, peri‐SWS areas, and FCD II lesion areas (non‐SWS). **** *p* < 0.0001, ordinary one‐way ANOVA. Error bars indicate SEM. H) Comparison of layer 1–2 vascular diameter distribution among SWS lesion areas, peri‐SWS areas, and FCD II lesion areas (non‐SWS).

To profile cells within SWS brain lesions, we obtained malformed areas (i.e., the SWS sample) and peri‐lesion areas (i.e., the control sample) from SWS patients who underwent neurosurgery for lesion resection (**Table** **1**). These patients had refractory epilepsy, and anti‐seizure medications were not able to adequately control their seizures. To evaluate the abnormalities in SWS cerebrovasculature, patients with focal cortical dysplasia type II (FCD II) were incorporated, which has no reported vascular malformations.^[^ ^15^ ^]^ The results of hematoxylin and eosin (HE) staining show a significant increase in leptomeningeal and layer 1–2 vascular diameter and density of SWS compared with FCD II and peri‐SWS, while the peri‐SWS samples exhibit no salient alterations compared to FCD II (**Figure** **1**B–H). We collected 11 samples (6 SWS samples and 5 control samples, Table 1) from four SWS patients for single‐cell RNA sequencing (scRNA‐seq) using the 10× Genomics Chromium platform. Consequently, we generated a dataset of high‐quality transcriptomes from 119 446 cells for further exploration (including 56 698 cells from the control samples, Figure 1A).

**Table 1:** Clinical diagnosis and operation information of SWS patients’ samples (Note: 1Y2 M, 1 year 2 months).

No.	Age (Y/M)	Gender	Diagnose	Affected hemisphere	Seizure types	Operation
Patient 1	1Y2M	Female	SWS, Secondary epilepsy	Right	Focal seizure	Functional hemispherectomy
Patient 2	11M	Male	SWS, Secondary epilepsy	Left	Focal seizure	Functional hemispherectomy
Patient 3	3Y4M	Male	SWS, Secondary epilepsy	Left	Focal seizure	Focal resection
Patient 4	1Y1M	Female	SWS, Secondary epilepsy	Right	Focal seizure	Focal resection

### 2.2 Global Analysis Identifies the Cell Populations and Alterations during SWS Progression

**Figure 2:**
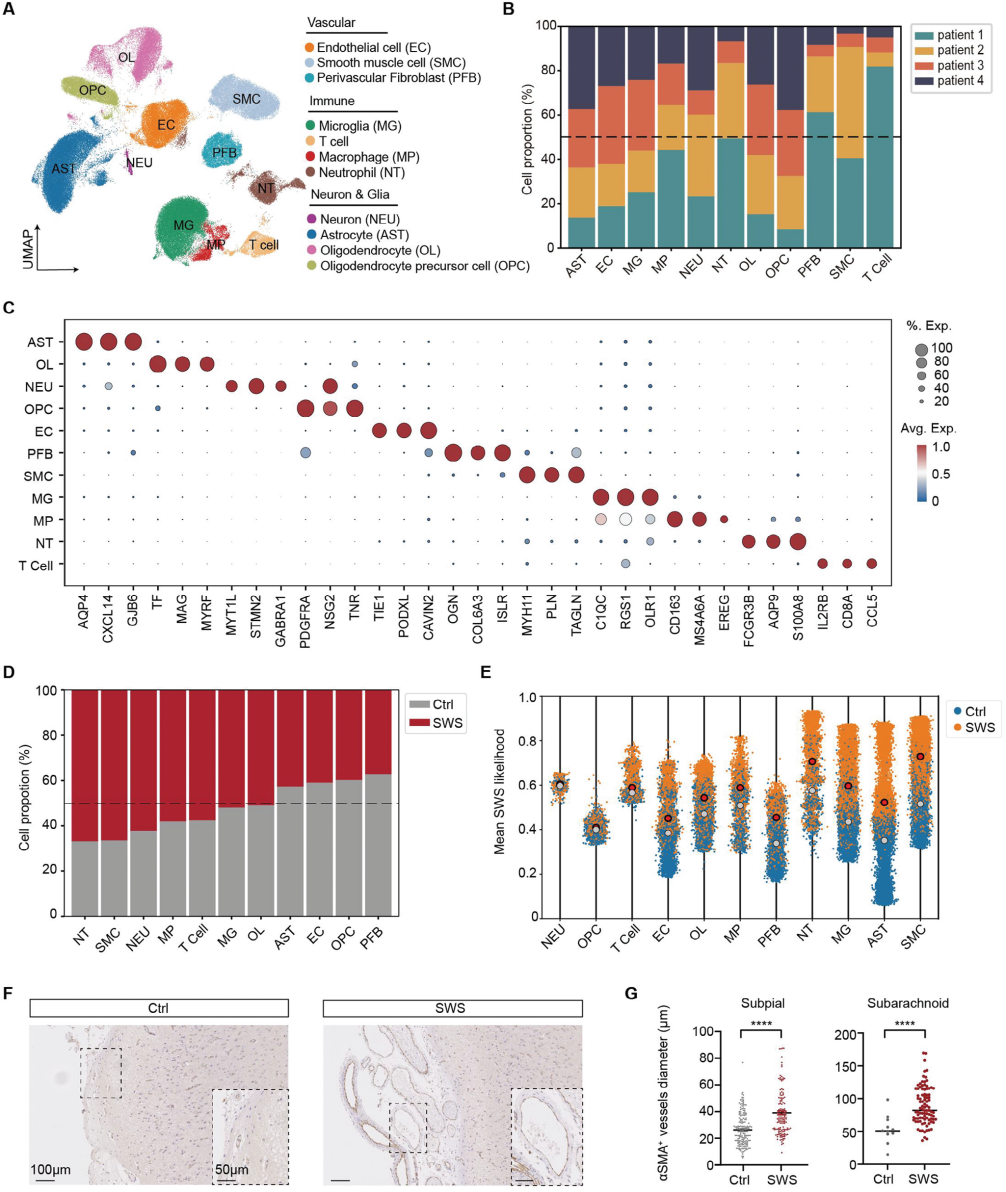
Identification of cell types within SWS lesions and peri‐lesion brain tissue from SWS patients. A) UMAP visualization showing 11 major cell types among the 11 SWS lesion and peri‐lesion samples from four SWS patients. Cell type abbreviations are defined in the key. B) The proportion of the 11 cell types derived from each patient based on their SWS and Ctrl samples. C) The top several gene markers for each major cell type. The size of each circle represents the percentage of cells expressing the target gene, and the color represents the average expression of the target gene. D) The proportion of each cell type isolated from SWS and control samples (SWS and Ctrl). Abbreviations: NT, neutrophil; SMC, smooth muscle cell; NEU, neuron; MP, macrophage; T Cell, CD8^+^ T cell; MG, microglial cell; OL, oligodendrocyte; AST, astrocyte; EC, endothelial cell; OPC, oligodendrocyte precursor cell; PFB, perivascular fibroblast. E) Jitter plot showing the likelihood of SWS associated with each cell type. The orange values represent the average likelihood of the cell being isolated from SWS samples, and the blue values represent the average likelihood of the cell being isolated from control samples. F) Immunostaining results show the expression of α‐SMA (labeling smooth muscle cells) in SWS and control (peri‐lesion) tissue. Slice thickness, 10 µm. Insets show the representative regions from the lesion and control group. G) Quantification of the diameter of αSMA+ blood vessels in SWS lesion and peri‐lesion tissues. ****p < 0.0001; two‐tailed unpaired t‐test. All error bars indicate SEM.

Applying graph‐based Louvain clustering, we identified 11 major cell populations and annotated them based on gene expression patterns across different cell types (**Figures** **2**A, S1A,B, Supporting Information). These annotated cell populations can be categorized into three main cell types: vascular cells, immune cells (note: we included microglia in this group although they are also a glial cell type), and neurons and glial cells (Figure 2A). Using the expression of well‐established cell type‐specific marker genes,^[^ ^16^, ^17^, ^18^, ^19^, ^20^ ^]^ we identified endothelial cells (*TIE1*), smooth muscle cells (*MYH11*), perivascular fibroblasts (*OGN,COL1A1,LUM*), macrophages (*CD163*), T cells (*CD8A*), neutrophils (*S100A8*), microglia (*C1QC*), astrocytes (*AQP4*), oligodendrocytes (*MAG*), oligodendrocyte precursor cells (*PDGFRA*), and neurons (*MYT1L*) (Figure 2C and Figure S3A, Supporting Information).

We then used a MELD analysis^[^ ^21^ ^]^ to identify the most relevant cell populations in relation to SWS. Notably, we observed distinct cell populations with mostly non‐overlapping average likelihood values, particularly for smooth muscle cells and astrocytes (Figure 2E). To further validate the results from the MELD analysis, we conducted immunostaining using antibodies against αSMA to label smooth muscle cells, and GFAP to label astrocytes respectively, in tissue sections from SWS lesions and peri‐lesions. Our findings revealed structurally irregular blood vessels containing SMC components (Figure 2F and Figure S7A, Supporting Information). Furthermore, a significant increase in vascular diameters was observed in both the subpial mater and subarachnoid space in SWS lesions (Figure 2G). These staining results align with the cell proportion analysis from SWS samples, which indicated an increase in the number of SMCs in SWS lesions as compared to peri‐lesion areas (Figure 2D). As for the astrocytes, the results revealed altered morphology, characterized by aggregation toward malformed blood vessels and a decreased GFAP^+^ area in the SWS region compared to the peri‐SWS region (Figure S7A,B, Supporting Information). Single‐cell RNA sequencing analysis of astrocytes identified differential expression of activation‐associated genes (e.g., GFAP, VIM, and AQP4) between the SWS and peri‐SWS regions. These molecular changes align with the morphological alterations observed in the staining results and further indicate a transition of astrocytes from a resting state to an activated state in response to the SWS microenvironment (see Figure S7C, Supporting Information).

In summary, this integrated analysis underscores the cellular alterations associated with the progression of SWS, providing a comprehensive perspective on the specific contributions of various cell populations to the pathology of SWS lesions.

### 2.3 Vascular Cells Analysis Reveals the Distinct Subsets and Recognizes the Presence of Meningeal Fibroblasts in SWS

**Figure 3:**
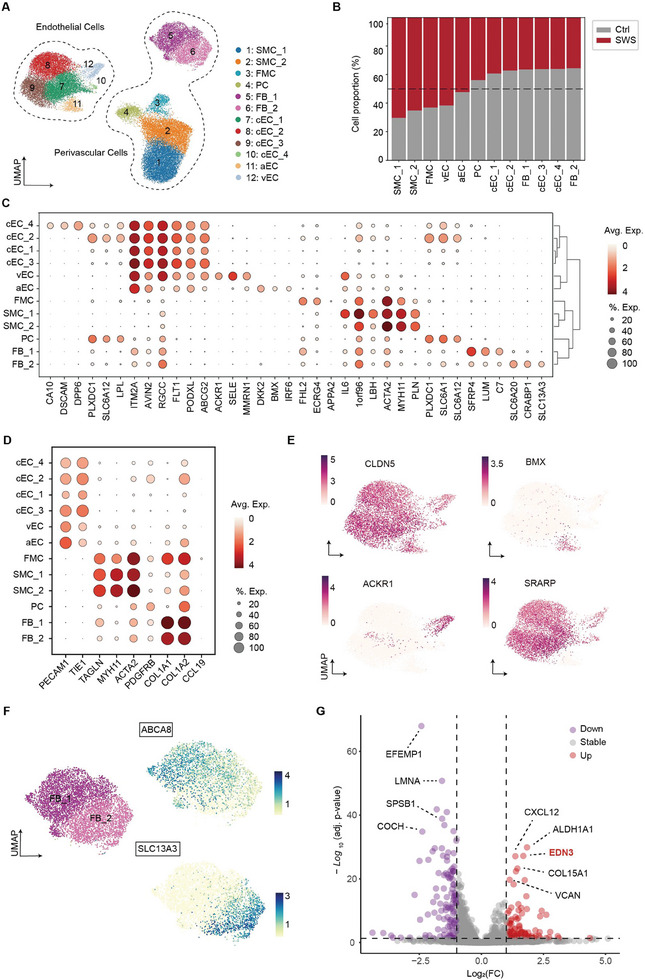
Cellular states of vascular cells during SWS progression. A) UMAP visualization depicting subpopulations of perivascular cells and endothelial cells. Abbreviations: SMC, smooth muscle cell; FMC, fibromyocyte; PC, pericyte; FB, fibroblast; cEC, capillary endothelial cell; aEC, arterial endothelial cell; vEC, venous endothelial cell. B) The proportion of vascular cell subtypes isolated from SWS and Ctrl (peri‐lesion) samples. C) The top several markers for each cell subpopulation in vascular cells. The size of the dot represents the percentage of cells expressing the marker gene, and the color represents the average expression of the marker gene. D) Dot plot presenting expression data for the classical markers of endothelial cells (*PECAM1* and *TIE1*), smooth muscle cells (*TAGLN, MYH11*, and *ACTA2*), pericytes (*PDGFRB*), fibromyocyte (*CCL19*), and fibroblasts (*COL1A1* and *COL1A2*) among each cell subpopulation in vascular cells. The size of the dot represents the percentage of cells expressing the marker gene, and the color represents the average expression of the marker gene. E) UMAP visualization depicting the expression of *CLDN5* (pan‐endothelial cell marker), *BMX* (arterial endothelial marker), *ACKR1* (venous endothelial marker), and *SRARP* (capillary endothelial marker). Darker colors mean the higher expression. F) UMAP visualization illustrating the cell states of perivascular fibroblasts (FB_1 and FB_2) and the expression of *ABCA8* and *SLC13A3*. Darker colors mean the higher expression. G) Volcano plot presenting the differentially expressed genes in FB_2 of SWS compared to the control group. Significance cutoff: |Log2(Fold change)| ≥ 1 and adj. *p*‐value ≤ 0.05. Upregulated genes highlighted: *CXCL12*, *ALDH1A1*, *EDN3*, *COL15A1*, and *VCAN*; downregulated genes highlighted: *EFEMP1*, *LMNA*, *SPSB1*, and *COCH*.

Leptomeningeal vascular malformation stands out as a primary pathological phenotype in the nervous system of SWS patients.^[^ ^6^ ^]^ A comprehensive exploration of cellular‐level alterations in SWS lesions is currently lacking. To provide a systematic understanding of the cellular changes within the SWS vasculature, we extracted vascular cells (endothelial cells, smooth muscle cells, and fibroblasts) and identified 12 distinct vascular cell subsets in a comprehensive analysis (Figure S3B, Supporting Information). These subsets encompassed endothelial cells, pericytes, two sub‐clusters of smooth muscle cells, fibromyocytes, and two sub‐clusters of fibroblasts. Building upon identified gene markers (*PECAM1* and *TIE1*) and recognizing the heterogeneity within endothelial cells, we further subdivided endothelial cells into six distinct sub‐clusters in our study: one arterial subtype, one venous subtype, and four capillary subtypes at the transcriptional level (**Figure** **3**A,D, Figure S2A,B, Supporting Information). Using *BMX*, *ACKR1*, and *RGCC* expression as distinguishing markers,^[^ ^16^ ^]^ we successfully annotated endothelial cells into arterial, venous, and capillary subtypes (Figure 3A,D). In terms of cell proportion, we observed a decline in all four sub‐clusters of capillary endothelial cells, whereas both arterial and venous endothelial cells showed an increase as compared to peri‐lesion control samples (Figure 3B). These findings suggest a substantial shift in the vascular composition within SWS malformations.

In addition to endothelial cells, perivascular cells including smooth muscle cells, pericytes, and fibroblasts play crucial roles in vascular repair, vascular homeostasis, and angiogenesis.^[^ ^22^ ^]^ Drawing on an earlier study,^[^ ^23^ ^]^ we distinguished pericytes from smooth muscle cells based on the high expression of *PLXDC1*, *SLC6A12*, and *SLC6A1* (Figure 3A,C). Within the category of smooth muscle cells, our results identified three subpopulations characterized by enriched expression of three markers: *TAGLN*, *MYH11*, and *ACTA2* (Figure 3A,D), which are established markers of smooth muscle cells. Notably, there was a distinct third cluster in smooth muscle cells with high expression of *FHL2*, *ECRG4*, and *PAPPA2*, setting it apart from the other two subclusters (SMC_1 and SMC_2 in Figure 3A,C). Previous research has indicated the presence of fibromyocytes originating from smooth muscle cells in vascular‐related diseases such as atheroprotective and arteriovenous malformations.^[^ ^24^ ^]^ Given the higher expression of *COL1A1* and *COL1A2*, lower expression of contractile proteins (*TAGLN* and *MYH11*) we annotated this cell population as fibromyocytes (Figure 3A,D). The more specific fibromyocyte markers (*CCL19*, *IGFBP5*), identified in a previous report,^[^ ^24^ ^]^ are more highly expressed in our annotated fibromyocytes compared to smooth muscle cells (SMC_1 and SMC_2). Moreover, the smooth muscle transcription factor MYOCD^[^ ^25^ ^]^ was barely expressed, suggesting that fibromyocytes are distinct from smooth muscle cells (Figure S3C, Supporting Information). To provide more evidence to define this fibromyocyte cell population, we performed additional analysis of the gene expression profiles. Compared with smooth muscle cells, fibromyocytes are enriched in extracellular matrix remodeling and signaling pathways characteristic of fibroblast‐like functions, such as extracellular matrix organization, while downregulating some smooth muscle‐associated traits, such as muscle contraction (Figure S3D,E, Supporting Information). When compared to fibroblasts, fibromyocytes are enriched for smooth muscle‐associated functions such as contraction and smooth muscle cell proliferation (Figure S3F,G, Supporting Information). These functional features align with the emerging concept of fibromyocytes as hybrid cells contributing to both structural and signaling roles within the vascular environment. In summary, these results suggest that a subset of cells undergoes a transition from smooth muscle cells to fibromyocytes in the brains of SWS patients. The contribution of this transition to the progression of SWS lesions requires further investigation.

From the analysis of cell proportions, the observed increase in the relative proportion of smooth muscle cells, fibromyocytes, and arterial and venous endothelial cells in SWS lesions indicates that different components of vessels, including arteries, veins, and capillaries undergo abnormal changes during the progression of SWS (Figure 3B).

In the CNS, fibroblasts are distributed in the meninges, perivascular spaces, and choroid plexus. In addition to the structural support that they provide, they may have specific functions in both the healthy and diseased human brain, although these remain largely uncharacterized. In our analysis, we identified cells characterized by high *COL1A1* and *COL1A2* expression as fibroblasts (Figure 3A,D). Within this fibroblast group, however, were two subgroups that exhibited distinct gene expression profiles. For instance, the first group (FB_1) expressed high levels of *SFRP4*, *LUM*, and *C7*, whereas the other fibroblast group (FB_2) exhibited higher expression of *SLC6A20*, *CRABP1*, and *SLC13A3* (Figure 3C). Building on known molecular definitions that SLC influx solute transporters are specifically expressed in meningeal fibroblasts and ABC efflux pumps are found mainly in perivascular fibroblasts,^[^ ^26^ ^]^ we identified *ABCA8* ^+^ fibroblasts (FB_1) as perivascular fibroblasts and *SLC13A3* ^+^ fibroblasts (FB_2) as meningeal fibroblasts (Figure 3F). These distinct expression patterns indicate potential differences in the metabolic roles of these fibroblast populations.

To illustrate the alterations and role of meningeal fibroblasts in SWS progression, we identified differentially expressed genes (DEGs) between SWS lesions and peri‐lesions. Among the upregulated genes, we observed increased *CXCL12* and *ALDH1A1* (Figure 3G), which are involved in angiogenesis,^[^ ^27^, ^28^ ^]^ and *COL15A1* and *VCAN* (Figure 3G), which play an important role in the extracellular matrix and provide support for the vascular wall.^[^ ^29^, ^30^ ^]^ These findings suggest that meningeal fibroblasts are critical players in vascular development and remodeling during SWS progression.

### 2.4 The EDN3⁺ Meningeal Fibroblasts as Potential Regulators during SWS Progression

**Figure 4:**
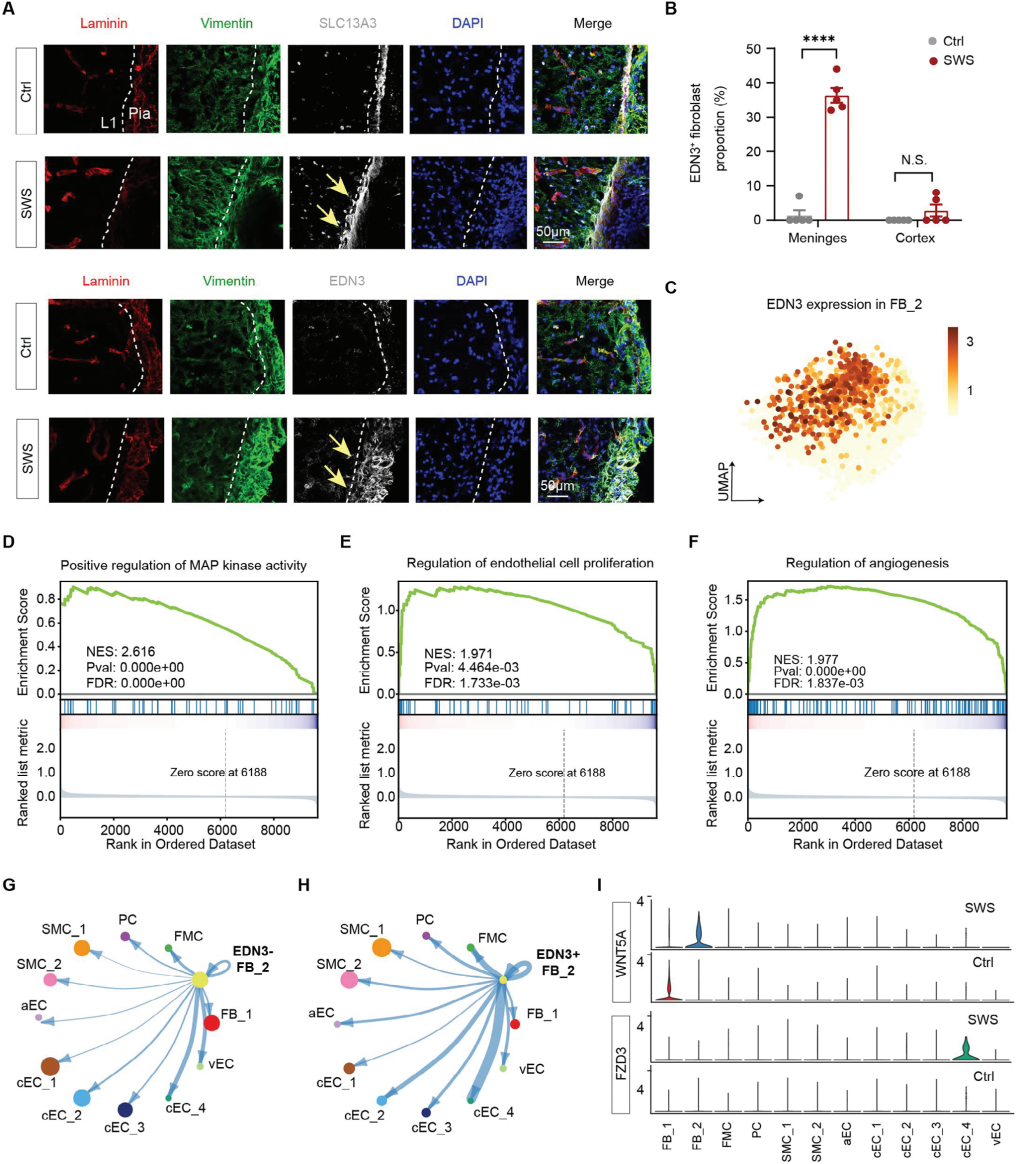
The alteration of *EDN3*⁺ meningeal perivascular fibroblasts during SWS disease progression. A) Immunostaining showing the expression of *SLC13A3* and *EDN3* in pial matter and layer 1 of the cerebral cortex from peri‐lesion (Ctrl) and SWS tissue. Laminin (red), vimentin (green), and EDN3/SLC13A3 (gray). Nuclei are stained with DAPI. The dashed lines indicate pial surface. Slice thickness, 50 µm. Scale bar, 50 µm. B) Quantification of *EDN*3^+^ fibroblasts in SWS and peri‐lesion tissue in different brain regions. ****p < 0.0001; N.S., not significant. Two‐tailed unpaired t‐test. Error bars indicate SEM. C) UMAP visualization depicting the expression of *EDN3* in FB_2. Darker colors mean the higher expression. D) Gene set enrichment analysis (GSEA) indicating the enrichment of positive regulation of MAP kinase activity in *EDN3* ^+^ cells from FB_2. E) Gene set enrichment analysis (GSEA) indicating the enrichment of Regulation of endothelial cell proliferation in *EDN3* ^+^ cells from FB_2. F) Gene set enrichment analysis (GSEA) indicating the enrichment of regulation of angiogenesis in *EDN3* ^+^ cells from FB_2. G) Circle plot presenting the signaling strength sent from *EDN3* ^−^ cells from FB_2 to other vascular‐related subclusters. The width of the edge is proportional to communication strength. H) Circle plot presenting the signaling strength sent from *EDN3* ^+^ cells from FB_2 to other vascular‐related subclusters. The width of the edge is proportional to communication strength. I) Violin plot presenting the expression level of *WNT5A* and *FZD3* (the ligand and receptor of ncWNT pathway) in each subgroup of SWS and Ctrl. The FB_2 in Ctrl is the *EDN3* ^−^ cells from FB_2, while FB_2 in SWS is the *EDN3* ^+^ cells.

Endothelin, which acts as a paracrine factor to regulate blood flow and vasoconstriction, is implicated in various disease processes involving the microvasculature, such as vascular hypertrophy.^[^ ^31^ ^]^ The endothelin system includes three ligands (EDN1, 2, and 3) and two receptors (EDNRA and EDNRB), highly expressed by mural and endothelial cells.^[^ ^31^ ^]^ We observed a significant increase in *EDN3*, one of the endothelin ligands, in SWS lesions. To validate this, we performed immunostaining of EDN3 and found a significant increase in *EDN3* expression in meningeal fibroblasts (*SLC13A3*⁺) in SWS tissue compared to peri‐lesion tissue (**Figures** **4**A,B and S6A, Supporting Information). To provide additional evidence for the increased *EDN3* expression in meningeal fibroblasts (*SLC13A3* ^+^) in SWS tissue compared to peri‐lesion tissue, we performed further analysis of our single‐cell data. Specifically, we calculated the *EDN3* expression in *SLC13A3* ^+^ cells, comparing its expression between the experimental group (SWS tissue) and the control group (peri‐lesion tissue). The results demonstrate a significant increase in *EDN3* expression in the experimental group (Figure S6B, Supporting Information), consistent with our previous finding. These results indicate that *EDN3*⁺ meningeal fibroblasts may be critical to SWS progression.

Our data shows that *EDN3* is not expressed in all meningeal fibroblasts (Figure 4C). Considering the potential association between *EDN3* expression in meningeal fibroblasts and SWS progression, we analyzed meningeal fibroblasts expressing *EDN3* (*EDN3*⁺ FB_2) as SWS‐related meningeal fibroblasts and used *EDN3* negative meningeal fibroblasts (*EDN3*⁻ FB_2) as controls. Gene set enrichment analysis (GSEA) revealed the enrichment of pathways such as positive regulation of MAP kinase activity (Figure 4D), regulation of endothelial cell proliferation (Figure 4E), and regulation of angiogenesis (Figure 4F) in *EDN3*⁺ meningeal fibroblasts, which are known to be activated in both SWS and other vascular malformations.^[^ ^12^, ^32^ ^]^

To validate the increased endothelial cell proliferation in the SWS region, we identified a subset of proliferative endothelial cells, accounting for 2.4% (Figure S5B,C, Supporting Information) which was characterized by the expression of proliferation‐related genes referenced from a previous study.^[^ ^33^ ^]^ The Ki67 (a proliferation marker) immunostaining staining also revealed Ki67‐positive endothelial cells in the SWS region while not in non‐SWS region (Figure S5A, Supporting Information). Then We quantified endothelial cell density in the SWS region and observed a significant increase compared to the non‐SWS region, suggesting enhanced angiogenesis in the SWS region (Figure S5D,E, Supporting Information). This analysis suggests that *EDN3*⁺ meningeal fibroblasts are closely associated with vascular malformation during SWS progression.

Cell–cell communication analysis is crucial for understanding the impact of disease states and the tissue microenvironment on intercellular communications and signal transduction. To explore the specific role of *EDN3*⁺ FB_2 during SWS progression, we focused on the overall information flow of a signaling network among vascular‐related subpopulations. Our analysis revealed that the outgoing interaction strength of *EDN3*⁺ meningeal fibroblasts is significantly enhanced (Figure S3A,B, Supporting Information), particularly in signaling from meningeal fibroblasts to a subcluster of capillary endothelium (Figure 4G,H).

The *EDN3*⁺ meningeal fibroblasts, as the subcluster for outgoing signals, show enriched signaling pathways such as CCL, ncWNT, L1CAM, and CXCL (Figure S3C, Supporting Information), which are associated with inflammation and angiogenesis.^[^ ^27^, ^34^, ^35^ ^]^ Notably, in the ncWNT (non‐canonical WNT) signaling pathway, an emerging player in cerebrovascular diseases, we observed increased ligand *WNT5A* in *EDN3*⁺ meningeal fibroblasts and its receptor *FZD3* in a subcluster of SWS capillary endothelium (Figure 4I). This observation is consistent with the increased signaling strength between these cells (Figure 4G,H).

The CCC analysis suggests that the ncWNT pathway plays an essential role in SWS development, with *WNT5A* in meningeal fibroblasts as a potential target to prevent progression. In summary, through GSEA and CCC analysis between *EDN3*⁺ and *EDN3*⁻ meningeal fibroblasts, we demonstrated the contribution of *EDN3*⁺ meningeal fibroblasts to SWS progression and identified *WNT5A* as a potential therapeutic target for SWS.

## 3 Discussion

Our scRNA‐seq analysis of lesioned and peri‐lesioned areas in the brains of SWS patients has provided valuable insights into the major cell populations that are involved, their states, molecular signals, and predicted cell‐to‐cell interactions. Notably, abnormalities were identified in several SWS‐related cell clusters, such as smooth muscle cells, including alterations in their relative proportions as compared to peri‐lesioned control samples.

Leptomeningeal vascular malformation stands out as the primary brain symptom of SWS.^[^ ^5^, ^36^ ^]^ Although SWS is fundamentally a disease of cerebrovascular malformation, it is difficult to assert that endothelial cells are the sole pathogenic factor. Previous studies have reported increased fibronectin expression in fibroblasts and brain tissue of SWS patients, which may contribute to blood vessel proliferation and neuronal degeneration.^[^ ^37^ ^]^ In our dataset, we identified a specific group of fibroblasts (FB_2) that express *SLC13A3* and are notably present in the meninges of SWS lesions. Moreover, our DEG analysis revealed the emergence of *EDN3* in meningeal fibroblasts in worsening SWS. To explicitly reflect the role of *EDN3*⁺ meningeal fibroblasts in SWS, we divided the meningeal fibroblasts into *EDN3*⁺ and *EDN3*⁻ categories. Comparing these two groups, we identified *WNT5A* as a ligand located on *EDN3*⁺ meningeal fibroblasts, which may contribute to SWS progression by sending more signals to a capillary endothelial subcluster (cEC_4) and activating the ncWNT signaling pathway.

An earlier study suggests that mutated GNAQ, encoding Gαq, is the candidate pathogenic gene for SWS,^[^ ^6^ ^]^ and vascular G protein‐coupled receptor signaling through Gαq includes endothelin receptors.^[^ ^38^ ^]^ From this perspective, endothelin may contribute to SWS vascular malformations, consistent with our results. *EDN3*, recognized as a cardiovascular peptide with significant implications in normal and pathological vasculature,^[^ ^39^ ^]^ is considered a therapeutic target. Our analysis identified EDN3⁺ meningeal fibroblasts as SWS‐related fibroblasts and illustrated their role and potential mechanism in SWS progression.

The WNT signaling pathways, consisting of the β‐catenin canonical pathway and the non‐canonical pathway, play a key role in cardiac development and angiogenesis.^[^ ^35^ ^]^ Previous studies have shown that *WNT5A*, the most prominent ligand in the non‐canonical pathway, primarily activates non‐canonical WNT signaling in the cardiovascular system.^[^ ^40^, ^41^ ^]^ In our analysis, we observed the activation of the non‐canonical pathway during SWS development and increased *WNT5A* expression in *EDN3*⁺ meningeal fibroblasts. However, further in vitro or in vivo research is required to validate the contribution of *WNT5A* and the ncWNT pathway in SWS progression.

SWS results in severely detrimental vascular malformations that affect children,^[^ ^5^ ^]^ yet treatment options for SWS have been lacking because of its poorly understood cellular and molecular mechanisms. Our study delineates the single‐cell transcriptional landscape of SWS, revealing cellular and molecular alterations in the cerebrovascular‐related cells during SWS progression. A more comprehensive understanding of SWS necessitates thorough investigations into its occurrence and development. We acknowledge limitations in scRNA‐seq technology, such as biases in cell capture. For instance, we observed fewer neurons in our samples and unanticipated deviations during cell isolation, potentially impacting relative cell proportions. Future validation with single‐cell nuclear sequencing technology is warranted. The high percentage of T‐cells observed in patient 1 likely reflects the heterogeneity among SWS patients, influenced by various factors such as immune system status, comorbidities, and recent immune responses. Ethical considerations preclude the availability of “normal” brain tissues as controls; instead, we used peri‐SWS lesions as controls. Additionally, more samples from SWS patients and reliable animal models are essential for further investigations.

## 4 Experimental Section

### Ethics Statement

Human brain tissue specimens and clinical data were collected from SanBo Brain Hospital (Capital Medical University, Beijing, China), with approval from the institutional ethics committee. Enrolled patients, who had been previously diagnosed with SWS and epilepsy, provided written informed consent for surgery and the use of anonymized data for scientific purposes. Experienced neurologists and neurosurgeons conducted routine assessments upon the patients' admission, collecting baseline characteristics and clinical information, including seizure history, angiomas, glaucoma, and other symptoms. Seizure types were classified according to the 2017 International League Against Epilepsy (ILAE) Classification. A multidisciplinary team consisting of neurosurgeons, neurologists, radiologists, electrophysiologists, and psychologists determined the surgical plan, including the type of surgery and the resection area. The diagnosis and surgical information for all human brain SWS tissues are summarized in Table 1.

### Isolation of Fresh Specimens

Isolation of fresh specimens involved the separation and excision of brain tissue, specifically the SWS lesion and peri‐SWS‐lesion brain tissue, into two approximately equal pieces (0.3–0.5 cm^3^, ≈300–500 mg). Each resulting brain tissue specimen was rinsed with pre‐cooled 4 °C saline, cut into six to eight small pieces, and transferred to a 1.5 mL tube with preservation solution (MACS Tissue Storage Solution, Miltenyi). The tubes were then placed in a cooler with ice for transportation. For vascular specimens, thicker vessels were visualized under a 5× magnification microscope. The vessels were gently held using forceps or vascular forceps while a scalpel and pair of forceps were used to peel away surrounding adherent tissue. The stripped vessels were stored in 1.5 mL tubes filled with 4 °C preservation solution before being sent out for scRNA‐seq.

### Single‐cell RNA sequencing

After harvesting, tissues were washed in ice‐cold RPMI1640 (Millipore, Sigma) and were dissociated using the Multi‐tissue dissociation kit 2 (Miltenyi Biotec). DNase treatment was carried out only for excessively viscous homogenates. Cell counts and viability were estimated using a fluorescence Cell Analyzer (Countstar Rigel S2) with acridine orange/propidium iodide (AO/PI) reagent (Miltenyi) after removing erythrocytes, and the decision to perform debris and dead cell removal (Miltenyi) was based on the results. Finally, fresh cells were washed twice in RPMI1640 and then resuspended at 1 × 10^6^ cells mL^−1^ in PBS and 0.04% bovine serum albumin.

Single‐cell RNA‐Seq libraries were prepared using single cell 3′ library preparation kit (SeekGene). Briefly, the appropriate number of cells was loaded into the flow channel of the chip. After removing the unsettled cells, a sufficient number of Cell Barcoded Magnetic Beads (CBBs) were pipetted into the flow channel. Next, cells in the MM chip were lysed to release RNA. Then, all CBBs were collected, and reverse transcription was performed at 37 °C for 30 min to label cDNA with the cell barcode on the beads. Further, Exonuclease I treatment was performed. Subsequently, barcoded cDNA on the CBBs was hybridized with a random primer. The resulting second strand DNA was denatured off the CBBs, purified, and amplified in a PCR reaction. The amplified cDNA product was then added to the sequencing adapter and sample index by indexed PCR. The indexed sequencing libraries were sequenced on the Illumina NovaSeq 6000.

### Immunostaining

Frozen human brain tissue from individuals with SWS was used for immunostaining. Tissue samples were sectioned at a thickness of 50 µm (Leica CM3050 S) and mounted on glass slides. The sections were then fixed with 4% paraformaldehyde in PBS at 4 °C for 30 min before being dehydrated through an ethanol series or air drying. Afterward, sections were blocked in a solution containing 3% BSA (Beyotime), 2% donkey serum (Jackson ImmunoResearch), and 0.3% Triton X‐100 (Aladdin) for 1.5 h at room temperature. Primary antibodies anti‐αSMA (1:300; ab5694, Abcam), anti‐GFAP (1:300; G3893, Merck), anti‐laminin‐2 (alpha‐2 chain, 1:300; ZRB1023, Merck), anti‐vimentin (1:300; AB10609472, Thermo Scientific), anti‐SLC13A3(1:300; 26184‐1‐AP, Proteintech) and anti‐endothelin 3 (1:300; AB2913459, Thermo Scientific) diluted in PBS were applied to the sections and incubated at 4 °C for 48 h. Detection of primary antibodies was achieved using the appropriate Alexa Fluor 488‐conjugated donkey anti‐rat IgG (1:1000; A21208, Thermo Scientific), Alexa Fluor 594‐conjugated goat anti‐rabbit IgG (1:1000; A32740, Thermo Scientific), and Alexa Fluor 647‐conjugated goat anti‐mouse IgG (1:1000, A32728, Thermo Scientific) secondary antibodies. The sections were then washed, and the nuclei were stained with DAPI. Co‐staining for anti‐SLC13A3 and anti‐EDN3 was performed using a multiple immunofluorescence kit (AFIHC023, Hunan Aifang Biological Technology, China) according to the manufacturer's protocol. Tyramide signal amplification technology was applied to enhance detection sensitivity. Imaging was carried out on a Leica TCS SP8 X confocal microscope with a 40× objective (Leica Microsystems).

### Image Processing and Quantification

Vessel diameter measurements were conducted on brain sections from SWS and peri‐region tissues stained with anti‐laminin‐2. For each sample, ≈200 blood vessel cross‐sections were analyzed from a total of five to seven areas of the cerebral cortex. Z‐stacks were captured using a Leica TCS SP8 confocal microscope (Leica Microsystems). Vessel diameter and length were manually measured using ImageJ software. For astrocyte morphological analysis, quantification was performed on 8‐bit confocal images obtained from samples visualized by immunostaining. The ROI manager in the ImageJ software was used to mark and measure the mean gray values of the GFAP^+^ segments of interest. The sum of all mean gray values per field was set as 100%, and the percentage of mean gray values per GFAP^+^ segment was calculated for each image. The results were graphed using GraphPad Prism (version 9.0) software.

### Single‐cell RNA‐sequencing Data Analysis

Raw scRNA‐seq reads underwent preprocessing using Cell Ranger (version 3.1.0) and were then aligned to the human reference genome (GRCh38 from UCSC) using the gene annotation file from GENCODE (human v32). The raw gene‐by‐barcode matrices generated by Cell Ranger were merged using Anndata, following instructions in the SCANPY tutorial.^[^ ^42^ ^]^ In the quality control step, cells with a minimum gene count of 300 and a minimum cell count of 50 with the expression of the gene of interest were retained. Subsequently, the count matrix was log‐transformed. After these preprocessing steps, a total of 119 446 cells were obtained for downstream analysis.

The MELD algorithm was used to estimate the differences in each cell under “normal” (i.e., control, or peri‐lesion region) and SWS conditions.^[^ ^21^ ^]^ Harmony,^[^ ^43^ ^]^ with default parameters, was used for batch removal, and Louvain clustering was performed with a resolution parameter set to 1.0. For annotation of major cell types, manual evaluation of classic marker genes was used (endothelial cell, *PECAM1* and *CLDN5*; astrocyte, *AQP4* and *SLC1A2*; macrophage, *CD83* and *CD163*; CD8**^+^** T cell, *CD8A*; neutrophil, *S100A8* and *FCGR3B*; oligodendrocyte precursor cell, *PDGFRA*; neuron, *GAP43* and *SYN2*; perivascular fibroblast, *DCN* and *COL1A2*; smooth muscle cell, *ACTA2* and *MYH11*; microglia, *TMEM119* and *GPR43*; oligodendrocyte, *MBP* and *MOBP*). A Wilcoxon test with tie correction was used to obtain ranked marker genes. Gene set enrichment analysis was performed using GSEAPY. Cell Chat^[^ ^44^ ^]^ was used for cell–cell communication analyses, following their recommended tutorials.

### Statistical Analysis

All quantified data were analyzed using GraphPad Prism (9.0). The difference between two groups was evaluated with a two‐tailed unpaired Student's t‐test, the difference between three groups was evaluated with ordinary one‐way ANOVA and the mean ± SEM along with individual data points are shown. Statistical significance was assessed by *p*‐values.

## Conflict of Interest

The authors declare no conflict of interest.

## Author Contributions

D.A., T.M., and X.L. contributed equally to this work. W.‐p.G. and Y.G. conceived and designed the project. Y.G. provided and organized tissue samples. X.L. participated in experimental design and pathological diagnosis. X.W., S.W., W.S., J.Z., M.X., and Z.C. conducted patient management and sample collection. X.Q. undertook pathological diagnosis and analysis of samples. F.L. performed sample preprocessing. D.A. and T.M. provided assistance in performing single‐cell RNA sequencing. D.A. and T.M. completed subsequent computational analysis. T.M. performed immunohistochemical staining and statistical analysis. T.M. and Z.B. drew diagrams. T.M., D.A., and W.‐p.G. wrote the manuscript with substantial input from all authors. W.S. and G.L. provided constructive suggestions for this study. W.‐p.G. and Y.G. supervised the study and provided reagents. All authors discussed, reviewed, and edited the manuscript.

## Supporting information



Supporting Information

## Data Availability

The data that support the findings of this study are available on request from the corresponding author. The data are not publicly available due to privacy or ethical restrictions.

## References

[1] Z. Zhao , A. R. Nelson , C. Betsholtz , B. V. Zlokovic , Cell 2015, 163, 1064. 26590417 10.1016/j.cell.2015.10.067 PMC4655822

[2] B. J. Andreone , B. Lacoste , C. Gu , Annu. Rev. Neurosci. 2015, 38, 25. 25782970 10.1146/annurev-neuro-071714-033835 PMC5729758

[3] J. Rustenhoven , C. Tanumihardja , J. Kipnis , Circ. Res. 2021, 129, 174. 34166075 10.1161/CIRCRESAHA.121.318173

[4] E. Storkebaum , A. Quaegebeur , M. Vikkula , P. Carmeliet , Nat. Neurosci. 2011, 14, 1390. 22030550 10.1038/nn.2947

[5] S. Yeom , A. M. Comi , Stroke 2022, 53, 3769. 36263782 10.1161/STROKEAHA.122.038585 PMC11062639

[6] M. D. Shirley , H. Tang , C. J. Gallione , J. D. Baugher , L. P. Frelin , B. Cohen , P. E. North , D. A. Marchuk , A. M. Comi , J. Pevsner , N. Engl. J. Med. 2013, 368, 1971. 23656586 10.1056/NEJMoa1213507 PMC3749068

[7] A. M. Day , C. E. McCulloch , A. M. Hammill , C. Juhász , W. D. Lo , A. L. Pinto , D. K. Miles , B. J. Fisher , K. L. Ball , A. A. Wilfong , A. V. Levin , A. J. Thau , A. M. Comi , J. I. Koenig , M. T. Lawton , D. A. Marchuk , M. A. Moses , S. F. Freedman , J. Pevsner , Pediatr. Neurol. 2019, 96, 30. 30853154 10.1016/j.pediatrneurol.2018.12.002 PMC7288445

[8] S. Wang , Q.‐Z. Liu , R. Zhao , X. Zhai , K. Zhang , L. Cai , S. Li , Z. Yang , Y. Shan , K. Ma , Y. Li , J. Hu , L. Sui , H. Cheng , X. Li , J. Su , M. Zhao , X. Wang , J. Zhou , M. Wang , T. Li , J. Zhang , S. Liang , G. Luan , Y. Guan , Neurology 2024, 103, e209525. 38875518 10.1212/WNL.0000000000209525 PMC11244739

[9] A. Dompmartin , C. J. M. van der Vleuten , V. Dekeuleneer , T. Duprez , N. Revencu , J. Désir , D. M. W. M. te Loo , U. Flucke , A. Eijkelenboom , L. Schultze Kool , M. Vikkula , L. Boon , Eur. J. Neurol. 2022, 29, 3061. 35715928 10.1111/ene.15452

[10] J. Thorpe , L. P. Frelin , M. McCann , C. A. Pardo , B. A. Cohen , A. M. Comi , J. Pevsner , J. Invest. Dermatol. 2021, 141, 685. 32771470 10.1016/j.jid.2020.03.978 PMC8483769

[11] R. Fjær , K. Marciniak , O. Sundnes , H. Hjorthaug , Y. Sheng , C. Hammarström , J. C. Sitek , M. D. Vigeland , P. H. Backe , A.‐M. Øye , J. H. Fosse , T. E. Stav‐Noraas , Y. Uchiyama , N. Matsumoto , A. Comi , J. Pevsner , G. Haraldsen , K. K. Selmer , Hum. Mol. Genet. 2021, 30, 1919. 34124757 10.1093/hmg/ddab144 PMC8522634

[12] S. Schrenk , L. J. Bischoff , J. Goines , Y. Cai , S. Vemaraju , Y. Odaka , S. R. Good , J. S. Palumbo , S. Szabo , D. Reynaud , C. D. Van Raamsdonk , R. A. Lang , E. Boscolo , Nat. Commun. 2023, 14, 1929. 37024491 10.1038/s41467-023-37516-7 PMC10079932

[13] U. M. Ayturk , J. A. Couto , S. Hann , J. B. Mulliken , K. L. Williams , A. Y. Huang , S. J. Fishman , T. K. Boyd , H. P. W. Kozakewich , J. Bischoff , A. K. Greene , M. L. Warman , Am. J. Hum. Genet. 2016, 98, 789. 27058448 10.1016/j.ajhg.2016.03.009 PMC4833432

[14] T. Funk , Y. Lim , A. M. Kulungowski , L. Prok , T. M. Crombleholme , K. Choate , A. L. Bruckner , JAMA Dermatol. 2016, 152, 1015. 27438697 10.1001/jamadermatol.2016.2365

[15] I. Blümcke , M. Thom , E. Aronica , D. D. Armstrong , H. V. Vinters , A. Palmini , T. S. Jacques , G. Avanzini , A. J. Barkovich , G. Battaglia , A. Becker , C. Cepeda , F. Cendes , N. Colombo , P. Crino , J. H. Cross , O. Delalande , F. Dubeau , J. Duncan , R. Guerrini , P. Kahane , G. Mathern , I. Najm , Ç. Özkara , C. Raybaud , A. Represa , S. N. Roper , N. Salamon , A. Schulze‐Bonhage , L. Tassi , et al., Epilepsia 2011, 52, 158. 21219302 10.1111/j.1528-1167.2010.02777.x PMC3058866

[16] F. J. Garcia , N. Sun , H. Lee , B. Godlewski , H. Mathys , K. Galani , B. Zhou , X. Jiang , A. P. Ng , J. Mantero , L.‐H. Tsai , D. A. Bennett , M. Sahin , M. Kellis , M. Heiman , Nature 2022, 603, 893. 35158371 10.1038/s41586-022-04521-7 PMC9680899

[17] J. D. Cahoy , B. Emery , A. Kaushal , L. C. Foo , J. L. Zamanian , K. S. Christopherson , Y. Xing , J. L. Lubischer , P. A. Krieg , S. A. Krupenko , W. J. Thompson , B. A. Barres , J. Neurosci. 2008, 28, 264. 18171944 10.1523/JNEUROSCI.4178-07.2008 PMC6671143

[18] Q. Li , B. A. Barres , Nat. Rev. Immunol. 2018, 18, 225. 29151590 10.1038/nri.2017.125

[19] K. Siletti , R. Hodge , A. Mossi Albiach , K. W. Lee , S.‐L. Ding , L. Hu , P. Lönnerberg , T. Bakken , T. Casper , M. Clark , N. Dee , J. Gloe , D. Hirschstein , N. V. Shapovalova , C. D. Keene , J. Nyhus , H. Tung , A. M. Yanny , E. Arenas , E. S. Lein , S. Linnarsson , Science 2023, 382, eadd7046. 37824663 10.1126/science.add7046

[20] M. Vanlandewijck , L. He , M. A. Mäe , J. Andrae , K. Ando , F. Del Gaudio , K. Nahar , T. Lebouvier , B. Laviña , L. Gouveia , Y. Sun , E. Raschperger , M. Räsänen , Y. Zarb , N. Mochizuki , A. Keller , U. Lendahl , C. Betsholtz , Nature 2018, 554, 475. 29443965 10.1038/nature25739

[21] D. B. Burkhardt , J. S. Stanley , A. Tong , A. L. Perdigoto , S. A. Gigante , K. C. Herold , G. Wolf , A. J. Giraldez , D. van Dijk , S. Krishnaswamy , Nat. Biotechnol. 2021, 39, 619. 33558698 10.1038/s41587-020-00803-5 PMC8122059

[22] E. Avolio , V. V. Alvino , M. T. Ghorbel , P. Campagnolo , Pharmacol. Ther. 2017, 171, 83. 27889329 10.1016/j.pharmthera.2016.11.002 PMC5345698

[23] B. D. Gastfriend , K. L. Foreman , M. E. Katt , S. P. Palecek , E. V. Shusta , J. Cereb. Blood Flow Metab. 2021, 41, 3052. 34027687 10.1177/0271678X211013700 PMC8756477

[24] E. A. Winkler , C. N. Kim , J. M. Ross , J. H. Garcia , E. Gil , I. Oh , L. Q. Chen , D. Wu , J. S. Catapano , K. Raygor , K. Narsinh , H. Kim , S. Weinsheimer , D. L. Cooke , B. P. Walcott , M. T. Lawton , N. Gupta , B. V. Zlokovic , E. F. Chang , A. A. Abla , D. A. Lim , T. J. Nowakowski , Science 2022, 375, eabi7377. 35084939 10.1126/science.abi7377 PMC8995178

[25] X. Long , R. D. Bell , W. T. Gerthoffer , B. V. Zlokovic , J. M. Miano , Arterioscler., Thromb., Vasc. Biol. 2008, 28, 1505. 18451334 10.1161/ATVBAHA.108.166066 PMC2574857

[26] A. C. Yang , R. T. Vest , F. Kern , D. P. Lee , M. Agam , C. A. Maat , P. M. Losada , M. B. Chen , N. Schaum , N. Khoury , A. Toland , K. Calcuttawala , H. Shin , R. Pálovics , A. Shin , E. Y. Wang , J. Luo , D. Gate , W. J. Schulz‐Schaeffer , P. Chu , J. A. Siegenthaler , M. W. McNerney , A. Keller , T. Wyss‐Coray , Nature 2022, 603, 885. 35165441 10.1038/s41586-021-04369-3 PMC9635042

[27] S. Cambier , M. Gouwy , P. Proost , Cell Mol. Immunol. 2023, 20, 217. 36725964 10.1038/s41423-023-00974-6 PMC9890491

[28] V. Ciccone , E. Terzuoli , S. Donnini , A. Giachetti , L. Morbidelli , M. Ziche , J. Exp. Clin. Cancer Res. 2018, 37, 311. 30541574 10.1186/s13046-018-0975-0 PMC6291966

[29] G. Martínez‐Nieto , R. Heljasvaara , A. Heikkinen , H.‐K. Kaski , R. Devarajan , O. Rinne , C. Henriksson , E. Thomson , C. von Hertzen , I. Miinalainen , H. Ruotsalainen , T. Pihlajaniemi , S.‐M. Karppinen , Int. J. Mol. Sci. 2021, 22, 9978. 34576139 10.3390/ijms22189978 PMC8467152

[30] S. Islam , H. Watanabe , J. Histochem. Cytochem. 2020, 68, 763. 33131383 10.1369/0022155420953922 PMC7649968

[31] E. R. Levin , N. Engl. J. Med. 1995, 333, 356. 7609754 10.1056/NEJM199508103330607

[32] L. Al‐Olabi , S. Polubothu , K. Dowsett , K. A. Andrews , P. Stadnik , A. P. Joseph , R. Knox , A. Pittman , G. Clark , W. Baird , N. Bulstrode , M. Glover , K. Gordon , D. Hargrave , S. M. Huson , T. S. Jacques , G. James , H. Kondolf , L. Kangesu , K. M. Keppler‐Noreuil , A. Khan , M. J. Lindhurst , M. Lipson , S. Mansour , J. O'Hara , C. Mahon , A. Mosica , C. Moss , A. Murthy , J. Ong , et al., J. Clin. Invest. 2018, 128, 1496. 29461977 10.1172/JCI98589 PMC5873857

[33] I. Tirosh , B. Izar , S. M. Prakadan , M. H. Wadsworth , D. Treacy , J. J. Trombetta , A. Rotem , C. Rodman , C. Lian , G. Murphy , M. Fallahi‐Sichani , K. Dutton‐Regester , J.‐R. Lin , O. Cohen , P. Shah , D. Lu , A. S. Genshaft , T. K. Hughes , C. G. K. Ziegler , S. W. Kazer , A. Gaillard , K. E. Kolb , A.‐C. Villani , C. M. Johannessen , A. Y. Andreev , E. M. Van Allen , M. Bertagnolli , P. K. Sorger , R. J. Sullivan , K. T. Flaherty , et al., Science 2016, 352, 189. 27124452 10.1126/science.aad0501 PMC4944528

[34] X. Lu , Z. Wang , D. Ye , Y. Feng , M. Liu , Y. Xu , M. Wang , J. Zhang , J. Liu , M. Zhao , S. Xu , J. Ye , J. Wan , Front. Pharmacol. 2021, 12, 765. 10.3389/fphar.2021.765768 PMC9163960 35668739

[35] Am G. , Pa D. , Angiogenesis 2002, 5, 1.12549854

[36] A. M. Comi , Lymphatic Res. Biol. 2007, 5, 257. 10.1089/lrb.2007.1016 18370916

[37] A. M. Comi , P. Hunt , M. P. Vawter , C. A. Pardo , K. G. Becker , J. Pevsner , Pediatr. Res. 2003, 53, 762. 12621118 10.1203/01.PDR.0000058921.54071.19

[38] C. D. Van Raamsdonk , K. R. Fitch , H. Fuchs , M. H. de Angelis , G. S. Barsh , Nat. Genet. 2004, 36, 961. 15322542 10.1038/ng1412 PMC7341985

[39] E. R. Levin , Am. J. Nephrol. 1996, 16, 246. 8739884 10.1159/000169004

[40] P. M. Bhatt , R. Malgor , Atherosclerosis 2014, 237, 155. 25240110 10.1016/j.atherosclerosis.2014.08.027 PMC4252768

[41] I. Akoumianakis , M. Polkinghorne , C. Antoniades , Nat. Rev. Cardiol. 2022, 19, 783. 35697779 10.1038/s41569-022-00718-5 PMC9191761

[42] F. A. Wolf , P. Angerer , F. J. Theis , Genome Biol. 2018, 19, 15. 29409532 10.1186/s13059-017-1382-0 PMC5802054

[43] I. Korsunsky , N. Millard , J. Fan , K. Slowikowski , F. Zhang , K. Wei , Y. Baglaenko , M. Brenner , P. Loh , S. Raychaudhuri , Nat. Methods 2019, 16, 1289. 31740819 10.1038/s41592-019-0619-0 PMC6884693

[44] S. Jin , C. F. Guerrero‐Juarez , L. Zhang , I. Chang , R. Ramos , C.‐H. Kuan , P. Myung , M. V. Plikus , Q. Nie , Nat. Commun. 2021, 12, 1088. 33597522 10.1038/s41467-021-21246-9 PMC7889871

