# Supplementary material for: Transcriptomic Profiling Unveils EDN3 + Meningeal Fibroblasts as Key Players in Sturge‐Weber Syndrome Pathogenesis
Source: Adv Sci (Weinh). 2025 Feb 8;12(17):2408888. doi: 10.1002/advs.202408888 (PMC12061316; doi:10.1002/advs.202408888)
Supplement: Supplementary file 1 — Supporting Information [file ADVS-12-2408888-s001.docx]

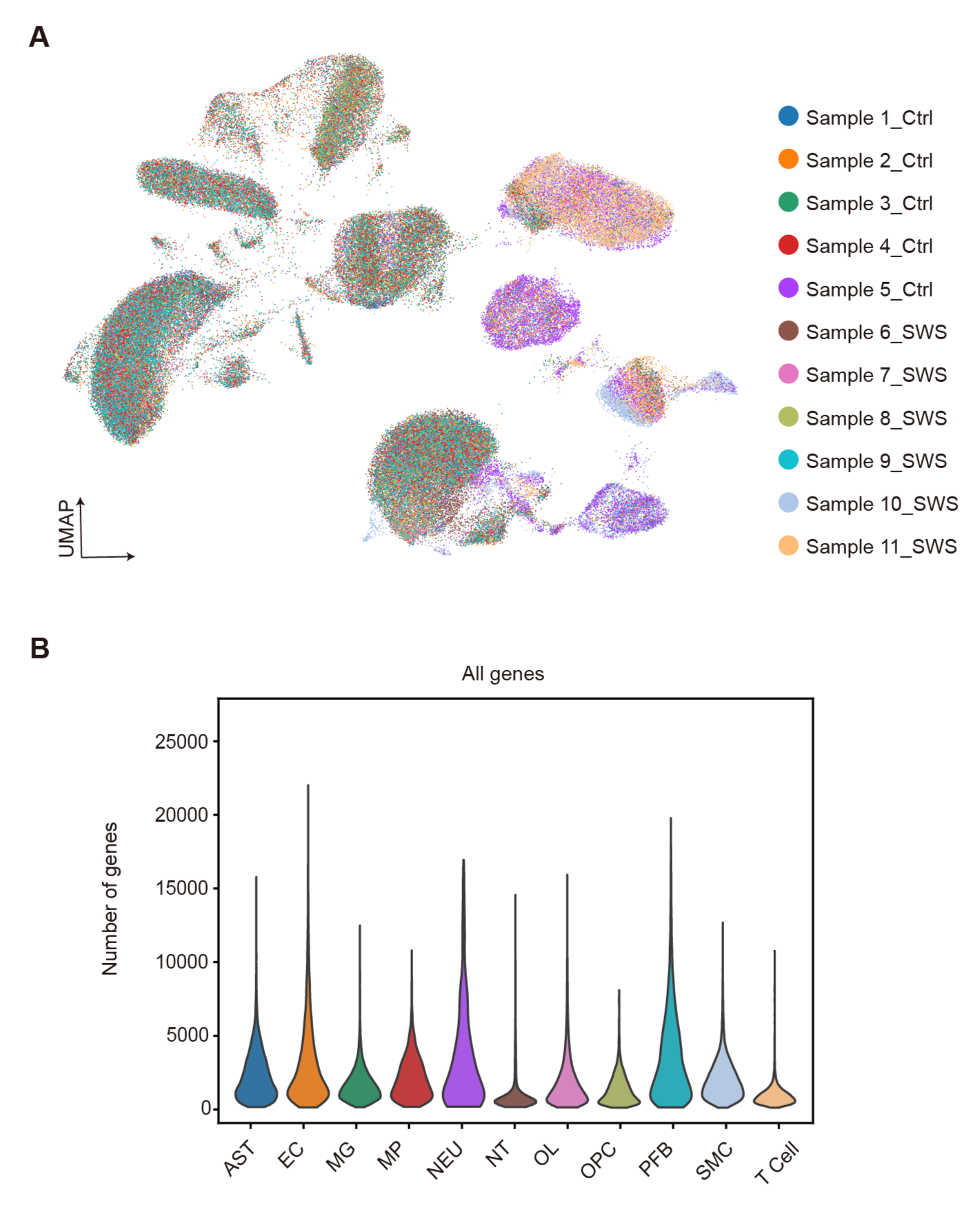
**Fig. S1 The whole cell populations basic information.**

1. UMAP visualization showing all cell populations from the 11 SWS samples.
2. Violin plots depicting the number of genes expressed across the 11 cell types after quality filtering. All gene indicated all of the genes we detected.

Abbreviations: AST, astrocyte; EC, endothelial cell; MG, microglial cell; MP, macrophage; NEU, neuron; NT, neutrophil; OL, oligodendrocyte; OPC, oligodendrocyte precursor cell; PFB, perivascular fibroblast; SMC, smooth muscle cell; T cell, CD8^+^ T cell.


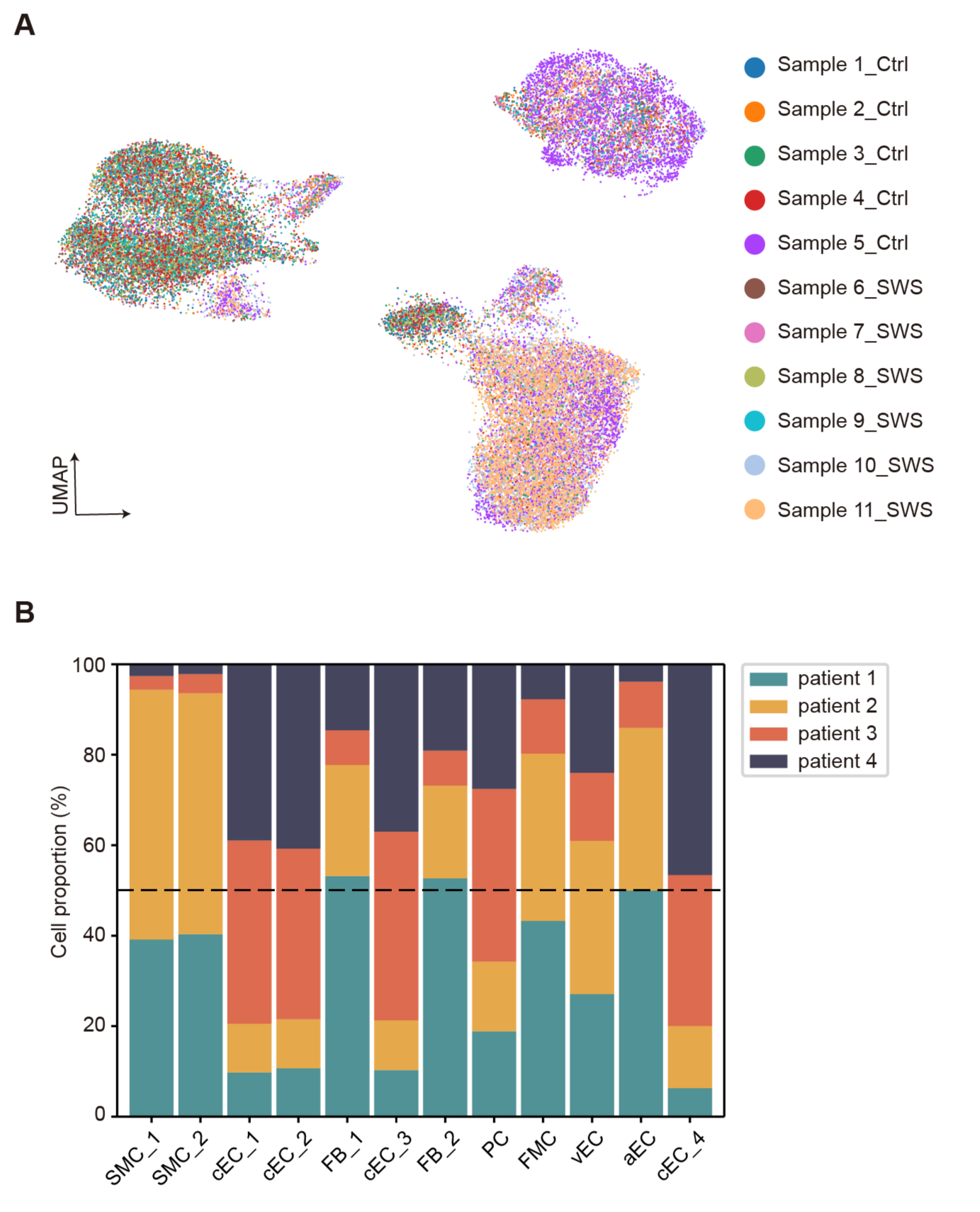
**Fig. S2 The vascular-related subpopulations basic information.**

1. UMAP visualization showing vascular cell populations from the 11 SWS samples.
2. The proportion of the 12 cell types derived from each patient based on their SWS and Ctrl samples.

Abbreviations: SMC, smooth muscle cell; FMC, fibromyocyte; PC, pericyte; FB, fibroblast; cEC, capillary endothelial cell; aEC, arterial endothelial cell; vEC, venous endothelial cell.

**
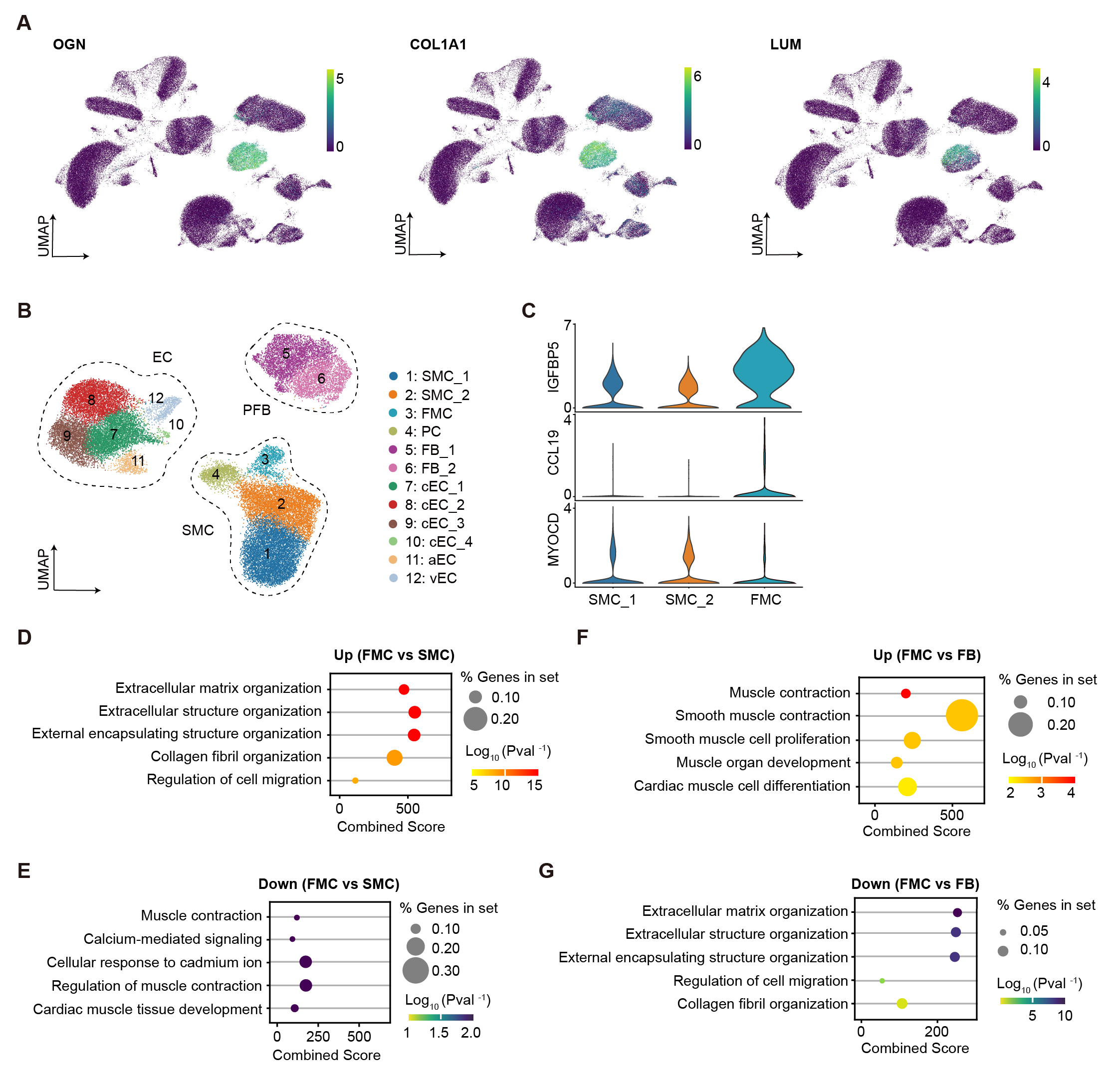
Fig. S3 The perivascular cells marker genes expression and functional analysis.**

1. UMAP visualization depicting the expression of classical fibroblast markers (*OGN, COL1A1,* and *LUM*) across 11 major cell types from four SWS patients. Blue indicates minimal expression, green represents intermediate expression, and yellow denotes high expression.
2. UMAP visualization showing the subpopulations of perivascular fibroblasts, smooth muscle cells, and endothelial cells annotated in Figure 2A. Abbreviations: SMC, smooth muscle cell; FMC, fibromyocyte; PC, pericyte; FB, fibroblast; cEC, capillary endothelial cell; aEC, arterial endothelial cell; vEC, venous endothelial cell.
3. A dot plot representing the five significantly upregulated (left) and downregulated (right) pathways in FMC compared to SMC, as determined by gene set enrichment analysis (GSEA). The size of each dot corresponds to the percentage of the gene list, while the color gradient indicates the level of significance.
4. A dot plot representing the five significantly upregulated pathways in FMC compared to SMC, as determined by gene set enrichment analysis (GSEA). The size of each dot corresponds to the percentage of the gene list, while the color gradient indicates the level of significance.
5. A dot plot representing the five significantly downregulated pathways in FMC compared to SMC, as determined by gene set enrichment analysis (GSEA). The size of each dot corresponds to the percentage of the gene list, while the color gradient indicates the level of significance.
6. Dot plot illustrating the five significantly upregulated pathways in fibromyocytes (FMC) compared to fibroblasts (FB), based on gene set enrichment analysis (GSEA). The size of the dots indicates the percentage of genes in the pathway, while the color gradient represents the level of statistical significance.
7. Dot plot illustrating the five significantly downregulated pathways in fibromyocytes (FMC) compared to fibroblasts (FB), based on gene set enrichment analysis (GSEA). The size of the dots indicates the percentage of genes in the pathway, while the color gradient represents the level of statistical significance.


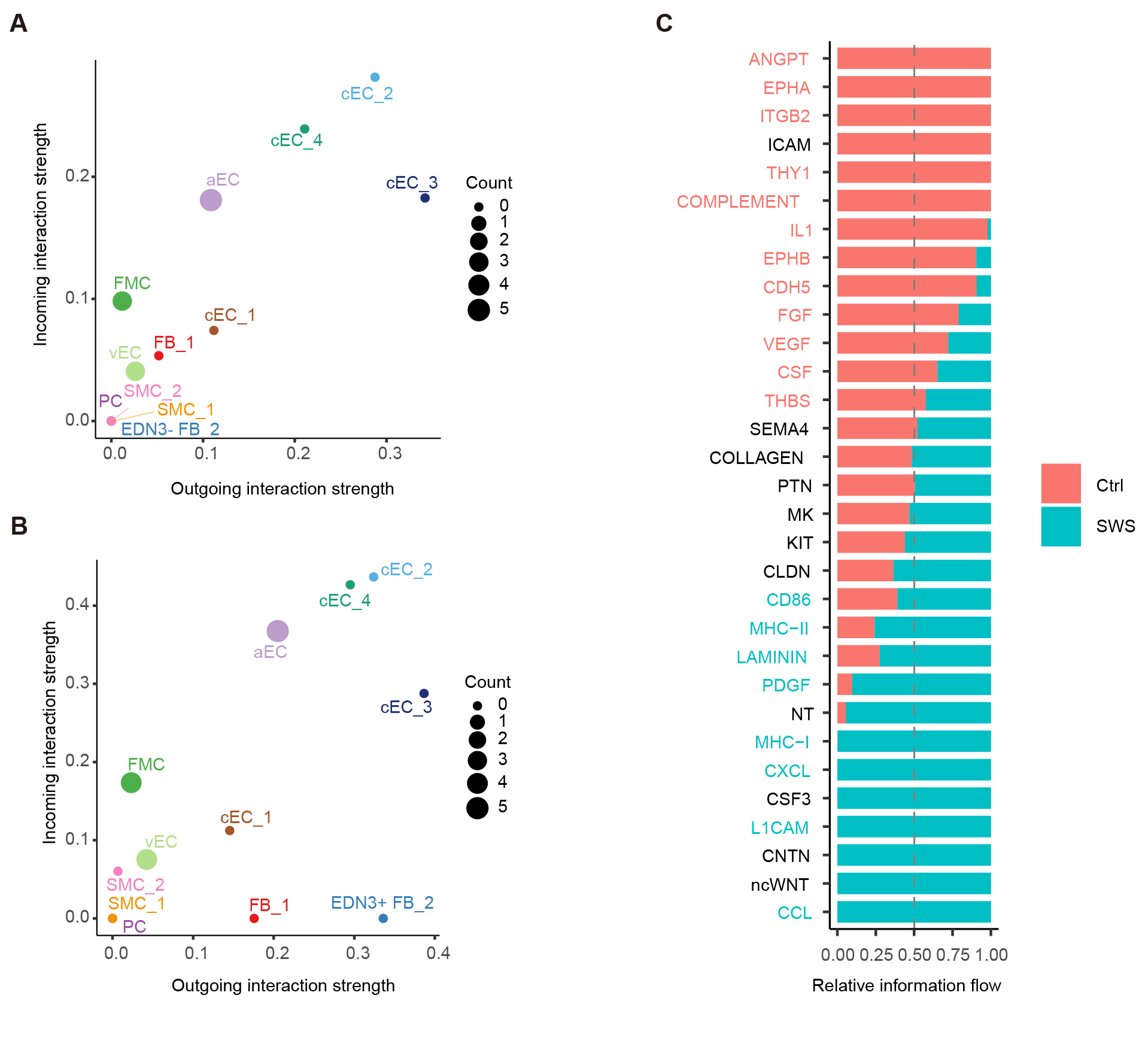
**Fig. S4 The alteration of cell-cell communication among vascular-related subclusters.**

1. Scatter plot showing the total communication probability associated with each cell group of control group. Dot size is proportional to the number of inferred links (both outgoing and incoming) associated with each cell group.
2. Scatter plot showing the total communication probability associated with each cell group of SWS group. Dot size is proportional to the number of inferred links (both outgoing and incoming) associated with each cell group.
3. All significant signaling pathways of meningeal perivascular fibroblasts, ranked based on their differences of overall information flow within the inferred networks between *EDN3*^+^ and *EDN3*^-^ FB_2. The top signaling pathways with higher enrichment in the control group are colored with salmon fonts, while those with higher enrichment in the SWS group are colored with teal fonts.

Abbreviations: SMC, smooth muscle cell; FMC, fibromyocyte; PC, pericyte; FB, fibroblast; cEC, capillary endothelial cell; aEC, arterial endothelial cell; vEC, venous endothelial cell.


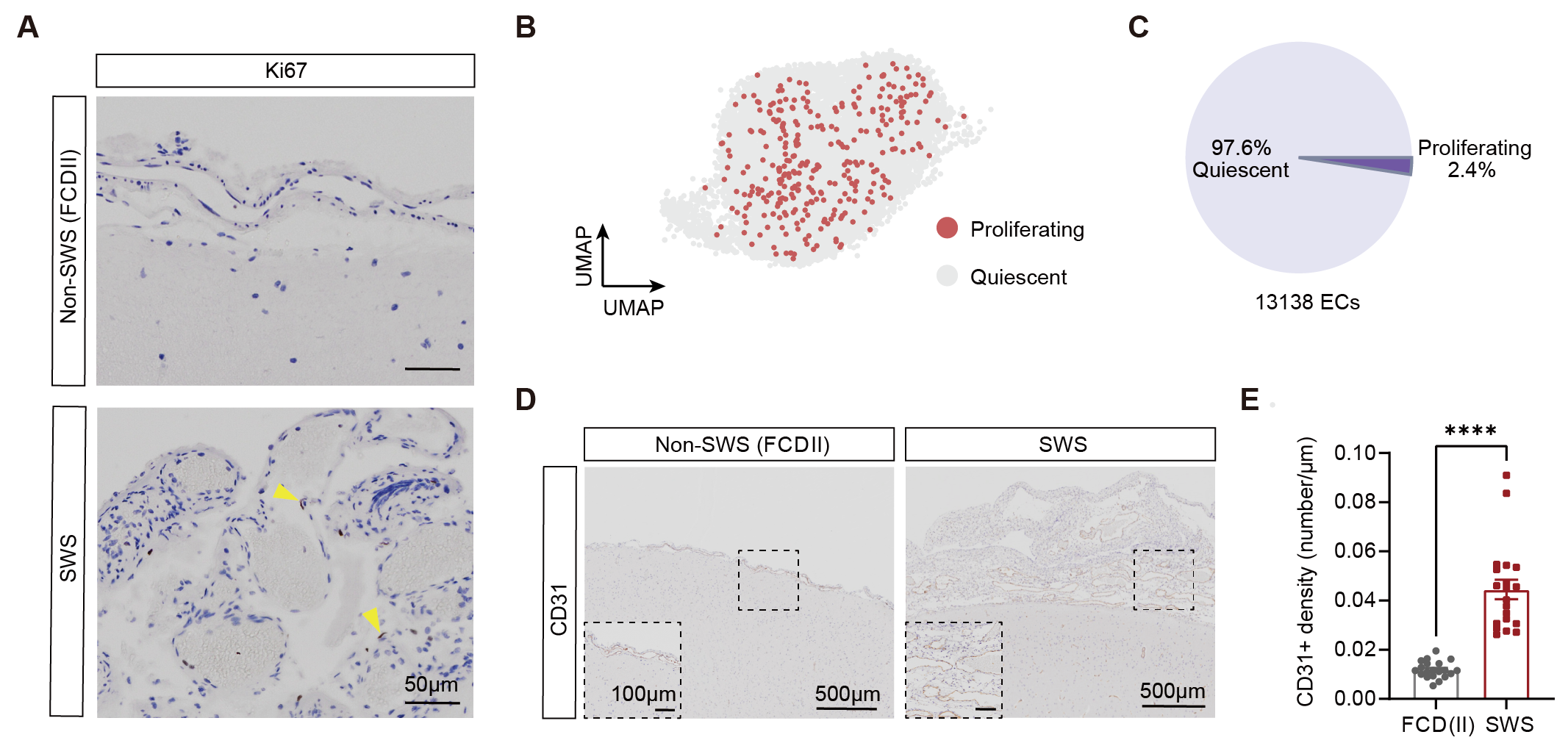
**Fig. S5 The endothelial cells proliferation in SWS region.**

1. Ki67 immunohistochemical staining showing Ki67-positive (Ki67^+^) cells in the SWS region. Arrowheads indicate Ki67^+^ cells with long nuclei, which are likely from endothelial cells. Scale bar: 50μm.
2. UMAP visualization depicting the proliferating and quiescent cells among the endothelial cluster. Red dots mean the cells expressing proliferating markers of S, G2 and M phase while grey dots in quiescent stage.
3. Pie chart showing the proportion of proliferating endothelial cells of the whole 13,138 endothelial cells.
4. CD31 immunohistochemical staining highlighting CD31^+^ cells in the non-SWS (FCD II) and SWS regions. Scale bar: 500μm.
5. Bar plot illustrating the density of CD3^+^ cells (cell number per vessel length) in the non-SWS (FCD II) and SWS regions. *****p* < 0.0001, two-tailed unpaired t-test. All error bars represent SEM.


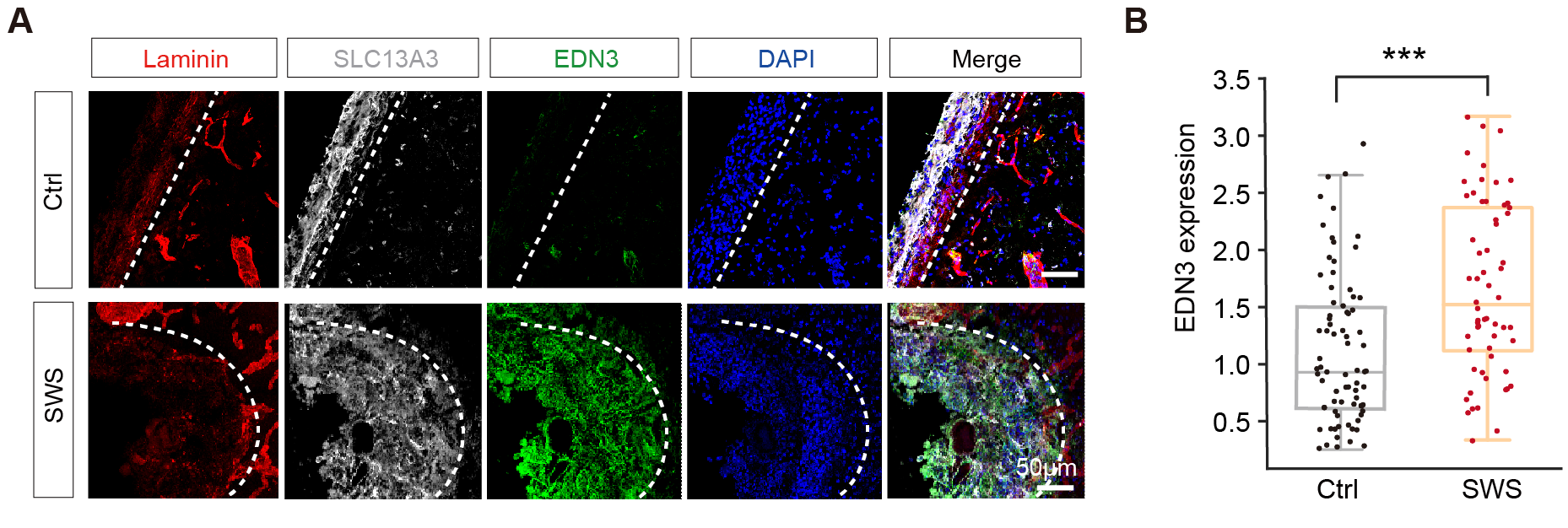
**Fig. S6 Co-staining and co-expression analysis of SLC13A3 and EDN3 in SWS and Ctrl (Peri-SWS) pial mater.**

1. Immunostaining showing the expression of SLC13A3 and EDN3 in the pial matter of the cerebral cortex from peri-lesion (Ctrl) and SWS tissue. Laminin (red), EDN3 (green), and SLC13A3 (gray). Nuclei were stained with DAPI. The dashed lines indicate the pial surface. Slice thickness: 50μm. Scale bar: 50μm.
2. The box plot showing the EDN3 expression level in SLC13A3^+^ meningeal fibroblasts from peri-lesion (Ctrl) and SWS tissue. Each dot represents one SLC13A3^+^ cell. *** *p* < 0.001, independent-samples t-test.


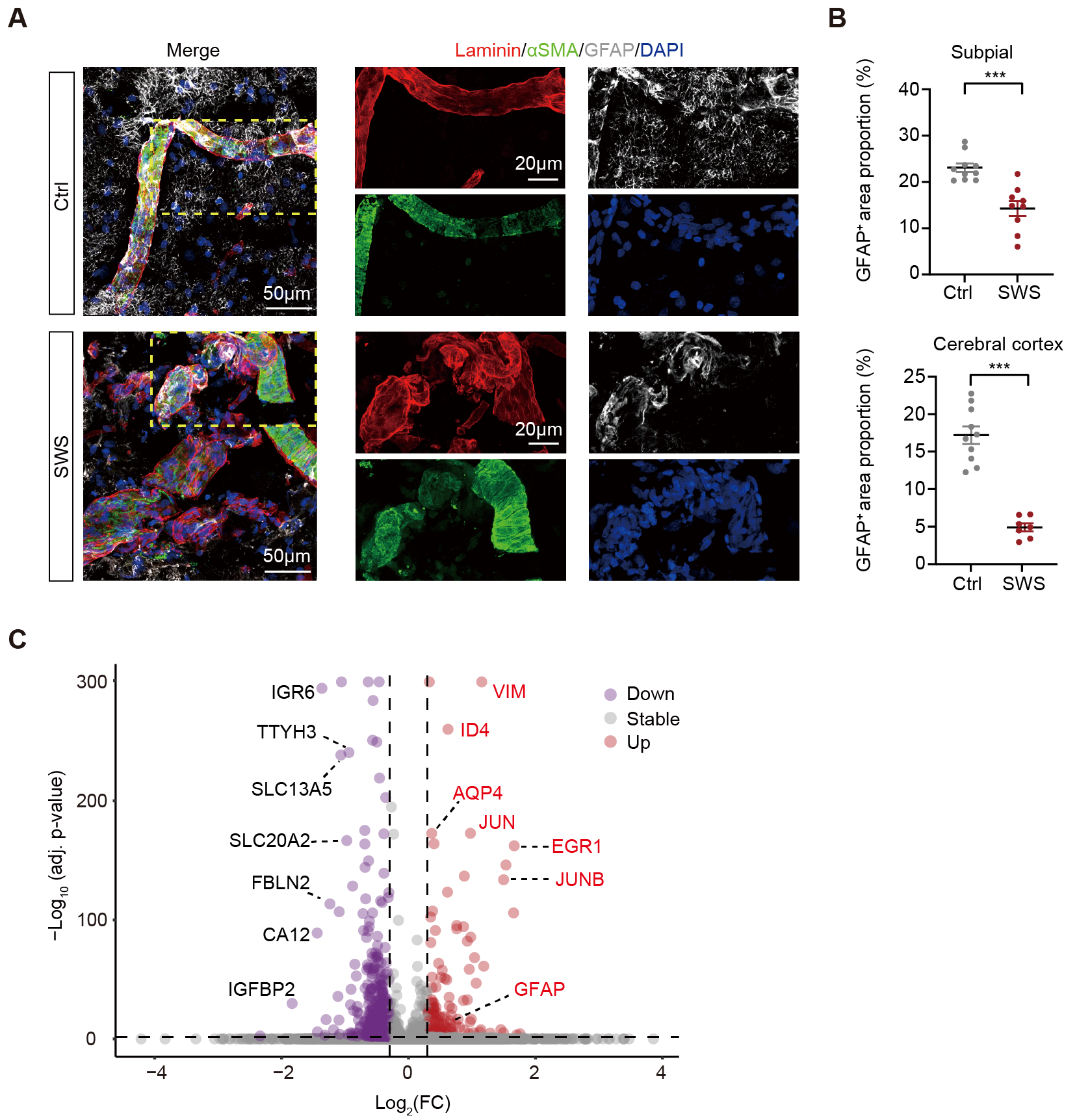
**Fig.S7 Astrocytes morphologic and gene alterations showing activation in SWS region.**

1. Immunostaining results using antibodies against Laminin (red, marking blood vessels), αSMA (green, marking smooth muscle cells), and GFAP (gray, marking astrocytes) in SWS and control (peri-lesion) tissue. The yellow dashed box highlights the region shown at higher magnification to the right. DAPI staining indicates cell nuclei. Slice thickness: 50μm. Scale bar: 20μm.
2. Quantification of the proportion of GFAP^+^ area in SWS lesion and peri-lesion tissues. ****p* < 0.001; two-tailed unpaired t-test. Error bars indicate SEM.
3. Volcano plot showing differentially expressed genes in astrocytes from SWS lesions compared to the control group. Significance thresholds: |Log2(Fold Change)| > 0.3 and adjusted *p*-value < 0.01. Highlighted upregulated genes: *VIM, ID4, AQP4, JUN, EGR1, JUNB*, and *GFAP.* Highlighted downregulated genes: *IGR6, TTYH3, SLC13A5, SLC20A2, FBLN2, CA12*, and *IGFBP2*.
